# Exposure to aircraft and road traffic noise and associations with heart disease and stroke in six European countries: a cross-sectional study

**DOI:** 10.1186/1476-069X-12-89

**Published:** 2013-10-16

**Authors:** Sarah Floud, Marta Blangiardo, Charlotte Clark, Kees de Hoogh, Wolfgang Babisch, Danny Houthuijs, Wim Swart, Göran Pershagen, Klea Katsouyanni, Manolis Velonakis, Federica Vigna-Taglianti, Ennio Cadum, Anna L Hansell

**Affiliations:** 1 MRC-PHE Centre for Environment and Health, Imperial College London, London, UK; 2 Cancer Epidemiology Unit, University of Oxford, Oxford, UK; 3 Centre for Psychiatry, Barts & the London School of Medicine, Queen Mary University of London, London, UK; 4 Department of Environmental Hygiene, Federal Environment Agency, Berlin, Germany; 5 National Institute for Public Health and the Environment, Bilthoven, the Netherlands; 6 Institute of Environmental Medicine, Karolinska Institute, Solna, Sweden; 7 Department of Hygiene, Epidemiology and Medical Statistics, Medical School, National and Kapodistrian University of Athens, Athens, Greece; 8 Laboratory of Prevention, Nurses School, National and Kapodistrian University of Athens, Athens, Greece; 9 Department of Clinical and Biological Sciences, University of Torino “San Luigi Gonzaga”, Orbassano, Italy; 10 Environmental Epidemiologic Unit, Regional Agency for Environmental Protection (ARPA), Piedmont Region, Grugliasco, Italy; 11 Public Health and Primary Care, Imperial College Healthcare NHS Trust, London, UK

**Keywords:** Air pollutants, Angina pectoris, Cardiovascular diseases, Myocardial infarction, Noise, Transportation, Stroke

## Abstract

**Background:**

Although a number of studies have found an association between aircraft noise and hypertension, there is a lack of evidence on associations with other cardiovascular disease. For road traffic noise, more studies are available but the extent of possible confounding by air pollution has not been established.

**Methods:**

This study used data from the Hypertension and Environmental Noise near Airports (HYENA) study. Cross-sectional associations between self-reported ‘heart disease and stroke’ and aircraft noise and road traffic noise were examined using data collected between 2004 and 2006 on 4712 participants (276 cases), who lived near airports in six European countries (UK, Germany, Netherlands, Sweden, Greece, Italy). Data were available to assess potential confounding by NO_2_ air pollution in a subsample of three countries (UK, Netherlands, Sweden).

**Results:**

An association between night-time average aircraft noise and ‘heart disease and stroke’ was found after adjustment for socio-demographic confounders for participants who had lived in the same place for ≥ 20 years (odds ratio (OR): 1.25 (95% confidence interval (CI) 1.03, 1.51) per 10 dB (A)); this association was robust to adjustment for exposure to air pollution in the subsample. 24 hour average road traffic noise exposure was associated with ‘heart disease and stroke’ (OR: 1.19 (95% CI 1.00, 1.41), but adjustment for air pollution in the subsample suggested this may have been due to confounding by air pollution. Statistical assessment (correlations and variance inflation factor) suggested only modest collinearity between noise and NO_2_ exposures.

**Conclusions:**

Exposure to aircraft noise over many years may increase risks of heart disease and stroke, although more studies are needed to establish how much the risks associated with road traffic noise may be explained by air pollution.

## Background

Cardiovascular diseases are the leading cause of mortality in Europe and worldwide. There is increasing evidence that environmental noise may increase the risks of cardiovascular diseases and hypertension [1]. Studies on the non-auditory effects of aircraft noise have established an association between exposure to aircraft noise and hypertension [2-7] but surprisingly few studies have examined heart disease or stroke and the overall evidence for an association could be described as tentative because of the small percentages exposed to high noise levels in these studies [8-10]. There have been more studies reporting on associations between heart disease or stroke and exposure to road traffic noise. These have shown an increased risk of myocardial infarction (MI) [9-15] but some studies have focused only on men [11,12] or found a significant association only in those who had not moved in 10 years [12] or only in those without exposure to other sources of noise [13].

Road traffic is a source of both noise and air pollution and, since air pollution has also been found to be associated with cardiovascular diseases in studies of long-term exposures, concerns have been raised about mutual confounding [16-18]. However the evidence for confounding of noise associations with heart disease or stroke by air pollution is uncertain. In five studies published to date, four studies showed an independent association between cardiovascular disease or stroke and road traffic noise after adjustment for air pollution [9,10,13,14] but one study found the effect of road traffic noise was confounded by air pollution [15].

This paper reports findings of the HYENA project (Hypertension and Exposure to Noise near Airports), a multi-centre cross-sectional study, which is one of the largest studies to investigate noise exposure in populations living near airports. Previous findings of this project have demonstrated an association between noise and cardiovascular disease risk factors [2,19-21]. The results are consistent with the hypothesis that noise exposure provokes a stress response causing a release of stress hormones, which in turn affect factors such as blood pressure and heart rate and thus cardiovascular disease risk [21-23]. It was therefore the aim of this study to investigate whether there was an association between exposure to aircraft noise or road traffic noise and heart disease and stroke. A secondary aim was to examine if any association between noise and heart disease and stroke was confounded by air pollution exposure, given the mutual sources.

## Methods

The HYENA cross-sectional survey has been described in detail elsewhere [2,24]. Briefly, it collected data between 2004 and 2006 on 4,861 adults (2404 men, 2457 women) aged 45–70 years who had lived at least five years (three years in the Greece sample) near seven European airports: London’s Heathrow, Amsterdam’s Schiphol, Stockholm’s Arlanda and Bromma, Milan’s Malpensa, Berlin’s Tegel and Athens’ Elephtherios Venizelos. Stratified random sampling using noise maps ensured participants were exposed to a range of noise levels from less than 50 A-weighted decibels (dB(A)) to greater than 60 dB(A) [24]. Across different noise exposure categories, the participation rates did not vary greatly: with response rates of 39, 45, and 45% for aircraft noise categories < 50, 50 to < 65, and ≥ 65 dB(A), respectively and response rates of 51, 42, 37% for road traffic noise [2]. However, participation rates did vary between countries, from approximately 30% in Germany, Italy, and the United Kingdom to 46% in the Netherlands, 56% in Greece, and 78% in Sweden [2]. Each participant was visited at home by staff who took clinical measurements and asked participants about doctor-diagnosed disease and about their lifestyles and home environment. The study was approved by ethical committees in all participating countries and informed written consent obtained. A subsample from three countries (UK, Netherlands and Sweden) was used where air pollution data of a comparable resolution to the noise data were available.

### Health outcomes

Participants were asked to report whether they had ever received a diagnosis from a doctor of a list of nine chronic diseases (high cholesterol, high blood pressure, angina pectoris, cardiac arrhythmia, myocardial infarction, stroke, diabetes, asthma, chronic bronchitis/emphysema and ‘other’ health problems) and to provide the year of first diagnosis for each condition by a medical practitioner, hospital or medical centre. The outcome of interest ‘heart disease and stroke’ was defined as a participant with a self-reported doctor’s diagnosis of angina pectoris, MI or stroke whilst living at their current address (if their year of diagnosis was equal to or greater than the year they moved into their current address). There were too few cases to allow for separate disease investigations.

### Exposure assessment

Annual average noise levels for 2002 were assigned to the home address of each participant using geographical information systems. All countries used the Integrated Noise Model (INM) to estimate aircraft noise exposure, except for the UK, which used the UK national model Ancon [2]. To estimate road traffic noise exposure, national noise models in each country were used [2]. The noise data were available at 1 dB(A) resolution, except for the UK road traffic noise data which were at 5 dB(A) resolution (midpoints of the 5 dB(A) classes were chosen for the continuous exposure variable) [2]. Noise that affects people’s ability to sleep might exert a different effect on their health, so aircraft noise indicators were chosen to represent daytime and night-time exposure: L_Aeq,16h_ (0700–2300) and L_night_ (2300–0700). L_Aeq,Th_ is the A-weighted equivalent continuous noise level over T hours, where A-weighting is used to approximate human hearing. However, information on road traffic flows at different time periods was not available in most of the study areas, so a 24 hour indicator L_Aeq,24h_ was chosen. Investigating night-time road traffic noise separately was not possible since L_Aeq,24h_ and L_night_ were highly correlated (overall r = 0.97) [2]. Uncertainty in the modelling of noise at low levels and lack of information on roads with low volumes of traffic meant that a cut-off value was introduced in each country based on a local assessment of the input data and noise model characteristics [2]. The highest local cut-off level was then applied to all data: assigning all values below to the cut-off level (35 dB(A) for daytime aircraft; 30 dB(A) for night-time aircraft; 45 dB(A) for road traffic) (Additional file 1: Figure S1). The spatial resolution was 250 m × 250 m for aircraft and 10 m × 10 m for road traffic noise.

Dispersion modelling of nitrogen dioxide (NO_2_) was used to estimate exposure to air pollution at the participants’ residence. A detailed account of the air pollution models is provided in the Additional file 1, page 2. Briefly, for the UK, modelled concentrations at a resolution of 20 m × 20 m were provided by King’s College London and derived using their London Emissions Toolkit and London Air Pollution Toolkit [25]. For the Netherlands, modelled concentrations were provided at 25 m × 25 m resolution using the EMPARA Luvotool model [26]. These modelled concentrations were mapped to participants’ home addresses using geographical information systems methods. For Sweden, concentrations at each HYENA participants’ address were provided by SLB-analys at 20 m × 20 m resolution, using the emission databases and dispersion models of Stockholm and Uppsala Air Quality Management Association [27].

### Statistical methods

Analyses were performed using Stata/IC 10.1 (StataCorp LP, College Station, TX). Odds ratios (ORs) and 95% confidence intervals (CIs) were used to estimate an association expressed per 10 dB(A) increment in noise using continuous exposure variables. For likelihood ratio tests (LRT), the null hypothesis was rejected if p < 0.05. For the main analysis without air pollution data, a hierarchical structure (random intercept) was specified to model possible differences between countries in the prevalence of ‘heart disease and stroke’ using multilevel logistic regression; a LRT to find the best-fitting model showed that including a random slope for country was not necessary.

Potential confounders considered for inclusion in the models were: age (continuous), sex (male, female), body mass index (BMI) (continuous), alcohol intake (teetotaller, 1–7 units per week, 8–14 units per week, > 14 units per week), physical activity (< once a week, 1–3 times a week, > 3 times a week), education (quartiles of number of years of education, standardised by each country’s mean number of years of education), smoking status (non-smokers, ex-smokers, 1–10 units per day, 11–20 units per day, > 20 units per day of cigarettes/pipes/cigars) and ethnicity (white, non-white). Confounders were included in the final regression model only if they caused a > 10% change in the coefficient of the exposure [28], which meant that only age, sex, BMI, education, ethnicity were included in the final models. The risk of heart disease and stroke is known to be higher for some ethnic groups [29]. Nearly a third of the UK sample was non-white but the other countries had few non-white participants, so a dichotomous variable was used. The two aircraft noise indicators (day and night) were not included in the same model because they were highly correlated (Spearman’s ρ = 0.82) (Additional file 1: Table S1).

Effect modification by age, sex, ethnicity and length of residence was investigated using stratified analyses and tests of interaction using the LRT. Categorical analyses in 5 dB(A) exposure categories were conducted to assess if any exposure-response relation was non-linear and tested using the LRT. 5 dB(A) categories were chosen rather than 10 dB(A) in order to detect differences between finer exposure categories. Associations with noise were also investigated for heart disease and stroke as separate outcomes as a sensitivity analysis.

For the subsample analysis with air pollution data, collinearity between NO_2_ air pollution and transport noise was investigated, given that both arise from the same sources. Three tests were used: Spearman’s ρ correlation coefficients; the correlation of the regression coefficients to show the correlation of the exposures in relation to ‘heart disease and stroke’; and the variance inflation factor (VIF), which is the inverse of 1–R^2^ and shows how much the variance of the coefficient estimate is inflated by multi-collinearity in the model [30]. The use of hierarchical models was rejected because there were less than five countries [31], so fixed effect logistic regression models were used. NO_2_ in Sweden had a different distribution compared to the other two countries (Additional file 1: Figure S2), so Sweden was investigated separately and a dummy variable for country was included in the combined sample of UK and Netherlands. The selection of confounders was repeated for the sample of three countries and this led to the following covariates being included in the final models: age, sex, education, ethnicity, BMI, physical activity, alcohol intake and smoking (which was measured in 3 categories (never, past, current)). To assess confounding of noise by air pollution, the percentage change in the coefficient of the noise exposure was calculated, once air pollution was included.

## Results

### Descriptive results

The analysis involved 4712 (276 cases) of the original 4861 HYENA participants, who had non-missing information on outcomes and confounders (see flow chart Additional file 1: Figure S3). The subsample analysis with both noise and air pollution data was conducted on 2401 participants (137 cases) with non-missing information from the original 2501 individuals (Additional file 1: Figure S3).

The average age of the participants was 53 years and 50% were male (Table 1). The prevalence of self-reported ‘heart disease and stroke’ in the HYENA population was 5.9%. The UK had the highest prevalence (8.8%) and Italy the lowest prevalence (3.6%) but 25 participants from Italy were excluded from the analysis because their year of diagnosis was missing. The distributions of noise by country overlapped but UK participants had the highest levels of aircraft noise and German participants the highest road traffic noise (Additional file 1: Figure S1, see also Additional file 1: Table S2 for noise exposure frequency distributions for the study population). The distributions of NO_2_ were similar in the UK and Netherlands but were over a much lower range in Sweden (Additional file 1: Figure S2).

**Table 1:** Participant characteristics, overall and by country, the HYENA study, 2004–2006

	**Overall**	**UK**	**Germany**	**Netherlands**	**Sweden**	**Greece**	**Italy**
No. of participants	4712	558	968	881	997	609	699
No. of cases of heart disease and stroke (%)	276 (5.9)	49 (8.8)	77 (8.0)	36 (4.1)	54 (5.4)	35 (5.7)	25 (3.6)
No. of cases of myocardial infarction^a^	133 (2.8)	14 (2.5)	46 (4.8)	19 (2.2)	29 (2.9)	14 (2.3)	11 (1.6)
No. of cases of angina pectoris^a^	144 (3.1)	34 (6.1)	28 (2.9)	21 (2.4)	26 (2.6)	22 (3.6)	13 (1.9)
No. of cases of stroke^a^	63 (1.3)	12 (2.2)	24 (2.5)	2 (0.2)	13 (1.3)	7 (1.1)	5 (0.7)
Daytime aircraft noise (dB(A))^b^							
Mean (SD)^b^	52 (9.5)	57 (9.7)	51 (10.7)	55 (6.3)	52 (8.6)	52 (7.2)	46 (10.3)
Range	35–76	35–76	35–74	38–74	35–66	37–66	35–70
Night-time aircraft noise (dB(A))							
Mean (SD)	41 (9.2)	49 (10.5)	40 (10.0)	42 (8.9)	40 (7.9)	42 (4.6)	35 (6.3)
Range	30–70	30–70	30–65	31–65	30–58	32–53	30–54
24 hour road traffic noise (dB(A))							
Mean (SD)	53 (7.5)	53 (5.3)	56 (8.1)	54 (7.1)	50 (5.3)	47 (4.9)	55 (9.1)
Range	45–77	45–75	45–73	45–74	45–71	45–69	45–77
Average NO2 (μg/m^3^)							
Mean (SD)	23.2 (1.3)	37 (3.5)	Not	32 (4.9)	8 (3.8)	Not	Not
Range	1–58	31–58	available	25–55	1–28	Available	available
Age							
Mean (SD)	58 (7.0)	59 (6.9)	57 (7.3)	58 (6.9)	57 (6.7)	58 (7.7)	57 (6.8)
Range	45–70	45–70	45–70	46–70	45–70	45–70	45–70
Gender (%)							
Male	49.6	51.8	48.2	49.2	51.8	45.7	50.8
Female	50.4	48.2	51.8	50.8	48.2	54.3	49.2
Years in education (%)							
1 Lowest quartile	24.5	21.9	13.2	18.6	23.5	40.4	37.5
2	25.3	26.0	55.4	35.3	15.9	3.9	2.6
3	25.8	32.3	16.0	26.6	35.9	12.6	30.5
4 Highest quartile	24.3	19.9	15.4	19.5	24.8	43.0	29.5
Ethnicity (%)							
White	95.7	71.5	98.5	98.4	98.6	99.8	99.6
Non-white	4.4	28.5	1.5	1.6	1.4	0.2	0.4
BMI							
Mean (SD)	27 (4.6)	28 (4.9)	28 (5.0)	27 (4.1)	26 (4.4)	28 (4.5)	26 (4.5)
Range	15–69	18–56	16–69	17–48	15–59	16–57	16–48
Physical activity (%)^c^							
<once/week,	32.5	46.5	26.5	15.4	31.4	32.0	53.5
1–3 times/week	22.9	22.9	23.2	27.5	25.4	17.3	18.2
>3 times/week	44.6	30.6	50.3	57.1	43.2	50.7	28.3
Smoking status (%)^c^							
Never	40.4	52.8	31.5	43.9	37.4	35.2	47.7
Past	34.5	34.6	37.7	32.5	43.8	23.3	28.5
Current	25.1	12.6	30.8	23.7	18.8	41.6	23.8
Alcohol consumption (%)^c^							
None	28.3	31.9	32.3	19.8	24.6	37.6	27.9
1–7 units/week	46.4	35.9	51.1	38.6	63.4	43.1	34.5
8–14 units/week	13.8	15.8	9.9	21.0	9.5	10.3	18.6
>14 units/week	11.5	16.3	6.6	20.6	2.5	9.1	19.1

^a^no. of cases of myocardial infarction, angina pectoris and stroke do not add up to no. of cases of heart disease and stroke because one participant could have more than one condition.

^b^dB(A), a measure of sound level in decibels A-weighted to approximate the typical sensitivity of the human ear; SD, Standard Deviation.

^c^some missing values were excluded: physical activity 0.3%; smoking status 0.5%; alcohol consumption 2.7%.

### Main noise analysis

**Figure 1:**
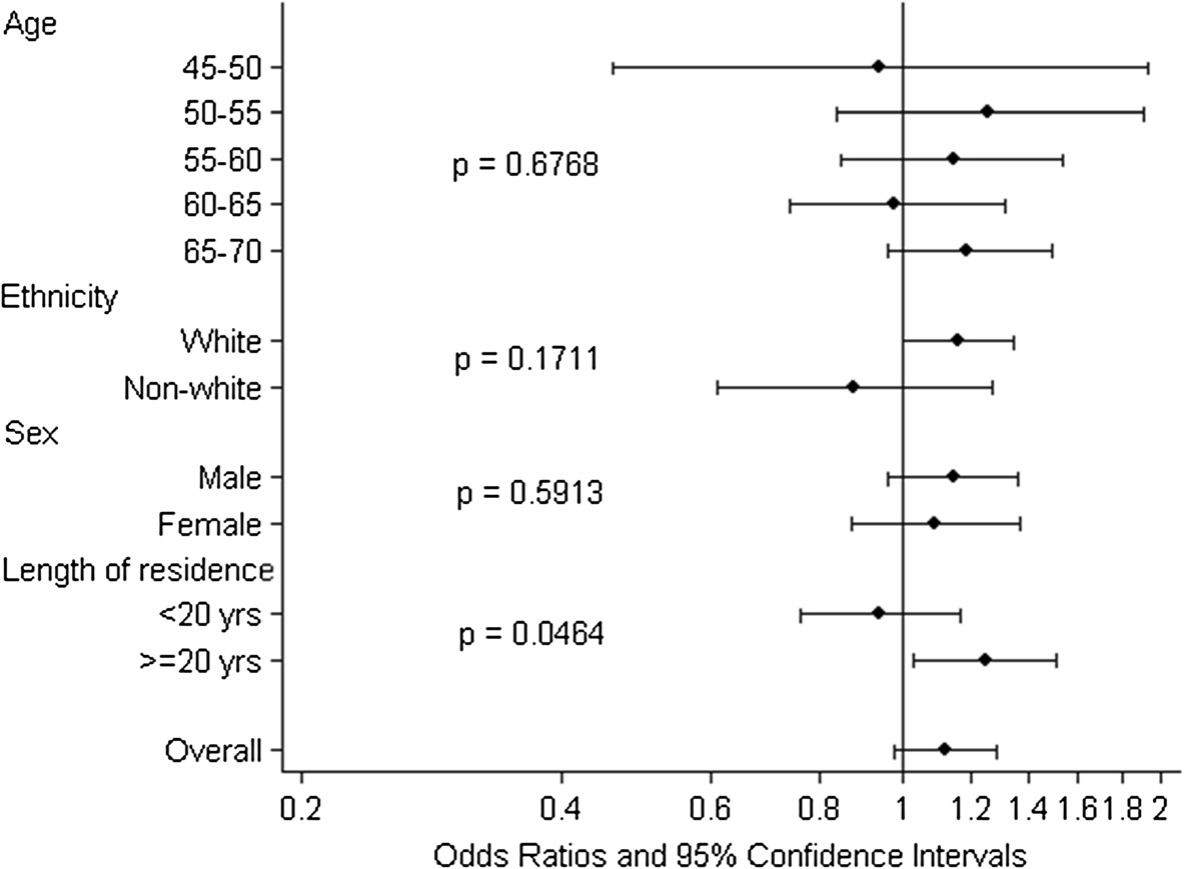
**Associations between ‘heart disease and stroke’ and night-time aircraft noise stratified by age, ethnicity, sex and length of residence.** Odds ratios and 95% confidence intervals. All models included a random intercept for country and were adjusted for age, sex, education, BMI, ethnicity and road traffic noise.

Night-time aircraft noise was statistically significantly associated with self-reported ‘heart disease and stroke’ in the crude model, but reduced and became non-significant after adjustment for confounders (Table 2). However, there was evidence for effect modification by length of residence (interaction p-value = 0.05) (Figure 1), with a significant association for those who had lived for 20 years or more at their current address (OR: 1.25 (1.03, 1.51)) (Table 2). Exposure to daytime aircraft noise was not associated with self-reported ‘heart disease and stroke’ (Table 2).

**Table 2:** Associations between ‘heart disease and stroke’ and daytime aircraft noise, night-time aircraft noise and 24-hour road traffic noise

**Heart disease and stroke Participants = 4712; Cases = 276**	**Daytime aircraft noise per 10 dB(A)**	**Night-time aircraft noise per 10 dB(A)**	**24 hr road traffic noise per 10 dB(A)**
Crude (exposure and random intercept^a^)	1.09 (0.95, 1.24)	1.18 (1.02, 1.35)	1.21 (1.02, 1.43)
Adjusted for age, sex, BMI, education, ethnicity^b^	1.05 (0.92, 1.21)	1.12 (0.97, 1.29)	1.18 (1.00, 1.41)
Adjusted for age, sex, BMI, education, ethnicity and other noise exposures^c^	1.06 (0.92, 1.21)	1.12 (0.98, 1.29)	1.19 (1.00, 1.41)
**≥ 20 years residence**			
**Heart disease and stroke Participants = 2236; Cases = 154**			
Crude (exposure and random intercept^a^)	1.17 (0.97, 1.40)	1.36 (1.10, 1.59)	1.20 (0.96, 1.51)
Adjusted for age, sex, BMI, education, ethnicity^b^	1.11 (0.92, 1.34)	1.24 (1.03, 1.50)	1.19 (0.94, 1.51)
Adjusted for age, sex, BMI, education, ethnicity and other noise exposures^c^	1.11 (0.92, 1.34)	1.25 (1.03, 1.51)	1.20 (0.95, 1.52)

Odds ratios and 95% Confidence Intervals.

^a^the hierarchical structure of each logistic regression model assumed a random intercept accounting for differences in heart disease and stroke prevalence between countries.

^b^age was measured as a continuous variable, sex as male or female, BMI as continuous, education as quartiles of years of education standardised by country means and ethnicity as white or non-white.

^c^both aircraft noise models were adjusted for road traffic noise and the road traffic noise model was adjusted for night time aircraft noise.

**Figure 2:**
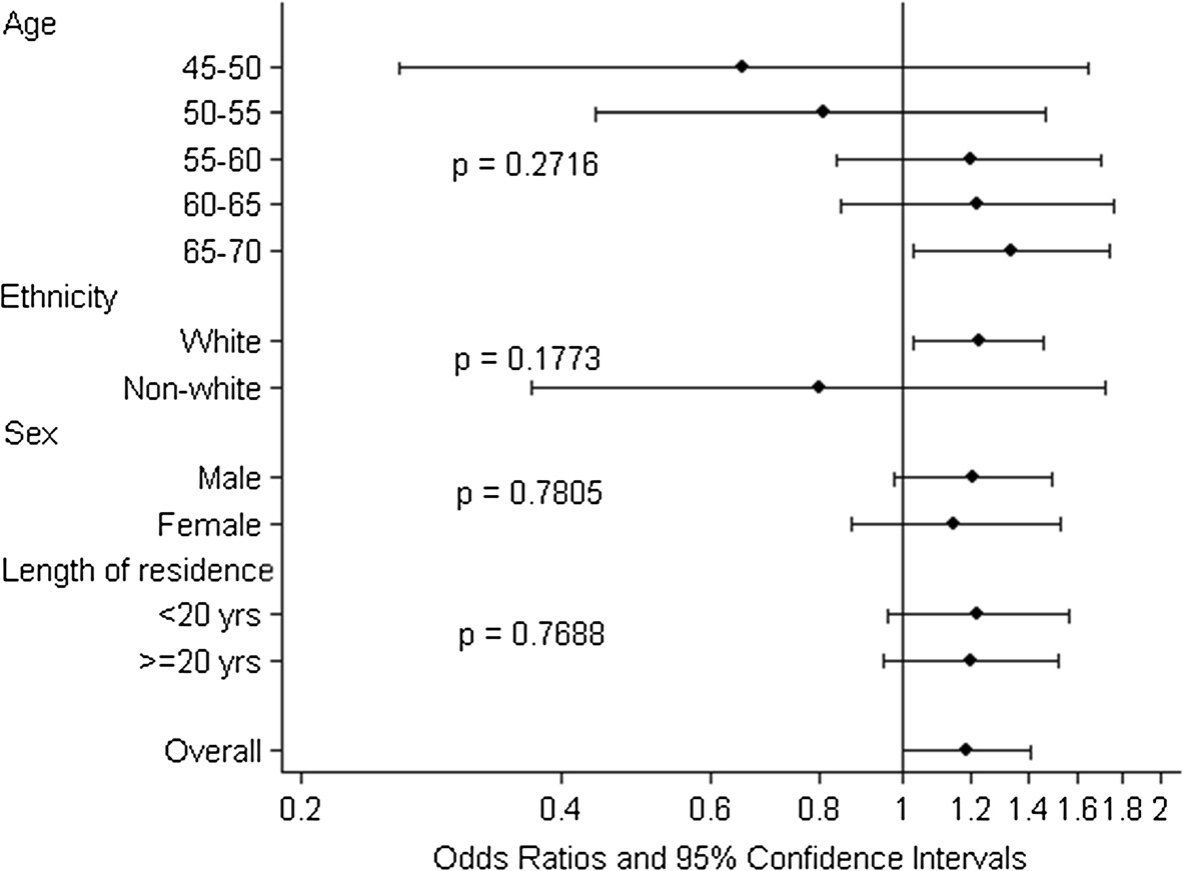
**Associations between ‘heart disease and stroke’ and 24-hour road traffic noise stratified by age, ethnicity, sex and length of residence.** Odds ratios and 95% confidence intervals. All models included a random intercept for country and were adjusted for age, sex, BMI, education, ethnicity and night-time aircraft noise.

There was an increase in odds of self-reported ‘heart disease and stroke’ in relation to road traffic noise that was stable after adjustment for confounders and exposure to night-time aircraft noise (OR: 1.19 (1.00, 1.41)) (Table 2). Adjusting for exposure to daytime aircraft noise, instead of night-time aircraft noise, did not change the results (data not shown). Effect modification by age or length of residence was not observed for road traffic noise, although a statistically significant association was found for participants aged 65–70 years (OR: 1.34 (1.03, 1.74)), whereas for lower age groups the associations were not statistically significant (Figure 2).

Categorical analyses did not suggest a threshold effect in the association between night-time aircraft noise and ‘heart disease and stroke’ (Additional file 1: Figure S4). An association between road traffic noise and ‘heart disease and stroke’ was found in the highest exposure category (≥ 65 dB(A): OR: 1.97 (1.19, 3.26)) compared to the lowest category (< 45 dB(A)), but the LRT did not provide evidence of non-linearity (Additional file 1: Figure S5). Separate analyses for heart disease only and for stroke only (Additional file 1: Table S3) showed similar estimates to the joint outcome in relation to all three exposures.

### Subsample analysis with air pollution exposure

There were weak correlations (both with bivariate Spearman’s ρ and correlations of estimated coefficients) between aircraft noise exposure and NO_2_ (Table 3). The correlations between road traffic noise and NO_2_ were moderate for the UK and Netherlands combined (ρ = 0.51) (and strong for Netherlands on its own (ρ = 0.74) (Additional file 1: Table S4)), thereby suggesting the potential for collinearity in the regression models. However, the VIF values were all below the suggested quantity of 2.5 as a cause for concern [30] and therefore collinearity in either the road traffic noise models or aircraft noise models was not thought likely to occur.

**Table 3:** Subsample analysis: Indicators of collinearity between noise and air pollution

**Collinearity indicators**^**a**^	**Daytime aircraft noise per 10 dB(A)**	**Night-time aircraft noise per 10 dB(A)**	**24 hr road traffic noise per 10 dB(A)**
**UK and Netherlands combined**			
**Participants = 1411**			
Spearman’s ρ	0.06*	0.11**	0.51**
Correlation of estimated coefficients	0.11	0.06	−0.50
Variance Inflation Factor	1.00	1.01	1.32
**Sweden**			
**Participants = 990**			
Spearman’s ρ	0.16**	−0.35**	0.35**
Correlation of estimated coefficients	0.15	0.43	−0.31
Variance Inflation Factor	1.03	1.21	1.11

Correlations of Exposure Variables (Spearman’s ρ), Correlation of Estimated Coefficients of Exposure Variables, Variance Inflation Factor, in UK and Netherlands combined and Sweden separately.

*P < 0.05.

**P < 0.0001.

^a^Only the two exposures were considered, adjustment was not made for other covariates.

In the UK and Netherlands combined sample, the associations between aircraft noise (daytime and night-time) and ‘heart disease and stroke’ rose slightly after adjustment for NO_2_ (Table 4). For participants who had lived for 20 years or more at the same address, the association between night-time aircraft noise and ‘heart disease and stroke’ was statistically significant after adjustment for NO_2_ (OR 1.43 (1.01, 2.01)) compared with (OR 1.33 (0.96, 1.84)) before adjustment for NO_2_ in the combined UK and Netherlands sample. The odds ratio in the Sweden sample was of the same magnitude but not statistically significant. Separate results for UK and Netherlands are shown in Additional file 1: Table S5.

**Table 4:** Subsample analysis: associations between ‘heart disease and stroke’ and noise adjusted for exposure to nitrogen dioxide

**Heart disease and stroke**	**Daytime aircraft noise per 10 dB(A)**	**Night-time aircraft noise per 10 dB(A)**	**24 hr road traffic noise per 10 dB(A)**
**UK and Netherlands combined**			
**Participants = 1411; Cases = 84**			
Crude (adjusted for country)	1.30 (0.98, 1.73)	1.22 (0.97, 1.53)	1.32 (0.92, 1.88)
Adjusted^a^	1.17 (0.86, 1.59)	1.14 (0.89, 1.45)	1.28 (0.88, 1.87)
Adjusted^a^ plus nitrogen dioxide exposure	1.24 (0.90, 1.71)	1.22 (0.95, 1.58)	0.93 (0.57, 1.53)
% change in coefficient (absolute value)	37%	54%	127%
**Sweden**			
**Participants = 990; Cases = 53**			
Crude	0.71 (0.54, 0.94)	1.01 (0.71, 1.43)	1.21 (0.74, 1.99)
Adjusted^a^	0.73 (0.54, 0.99)	0.88 (0.61, 1.27)	1.08 (0.64, 1.80)
Adjusted^a^ plus nitrogen dioxide exposure	0.74 (0.55, 1.01)	0.93 (0.62, 1.40)	0.99 (0.56, 1.73)
% change in coefficient (absolute value)	5%	46%	117%
**≥ 20 years residence**			
**UK and Netherlands combined**			
**Participants = 828; Cases = 52**			
Crude (adjusted for country)	1.30 (0.91, 1.87)	1.38 (1.02, 1.86)	1.51 (0.97, 2.36)
Adjusted^a^	1.19 (0.80, 1.77)	1.33 (0.96, 1.84)	1.49 (0.91, 2.43)
Adjusted^a^ plus nitrogen dioxide exposure	1.25 (0.82, 1.90)	1.43 (1.01, 2.01)	1.14 (0.61, 2.15)
% change in coefficient (absolute value)	29%	24%	67%
**Sweden**			
**Participants = 480; Cases = 35**			
Crude	0.93 (0.62, 1.38)	1.37 (0.87, 2.14)	0.95 (0.49, 1.86)
Adjusted^a^	1.02 (0.64, 1.63)	1.29 (0.77, 2.15)	0.80 (0.39, 1.62)
Adjusted^a^ plus nitrogen dioxide exposure	1.03 (0.64, 1.65)	1.36 (0.78, 2.37)	0.72 (0.32, 1.62)
% change in coefficient (absolute value)	26%	21%	39%

Associations expressed in odds ratios and 95% confidence intervals. % change in coefficient compares adjusted models before and after additional adjustment for nitrogen dioxide.

^a^Adjusted for age, sex, education, ethnicity, BMI, physical activity (< once/week, 1–3 times/week, > 3 times/week), smoking (never, past, current), alcohol intake (teetotal, 1–7 units/week, 8–14 units/week, >14 units/week; 1 unit = 10 ml pure ethanol). In addition, the aircraft noise models were adjusted for 24 hour road traffic noise and the road traffic noise model was adjusted for night-time aircraft noise.

The odds ratio for the association between road traffic noise and ‘heart disease and stroke’ in the subsample was higher than that found for the full six country sample but not statistically significant (Table 4). When adjustment was made for NO_2_, the odds ratio reduced to below 1 and the percentage change in the coefficient suggested confounding by NO_2_. A similar result was found for the Swedish sample, where a non-significant association with road traffic noise was reduced to null after adjustment for NO_2_ (Table 4).

In the UK and Netherlands sample, an increase of 10 μg/m^3^ of NO_2_ was associated with an OR of 1.85 (1.13, 3.02) when adjusted for all confounders except road traffic noise and an OR of 1.95 (1.03, 3.70) when additionally adjusted for road traffic noise (Additional file 1: Table S6). For Sweden, there was a non-statistically significant association for NO_2_ which did not change after adjustment for road traffic noise (Additional file 1: Table S6).

## Discussion

The aim was to examine the association between noise and ‘heart disease and stroke’ for residents exposed to varying levels of aircraft noise and road traffic noise around major airports across Europe. A statistically significant association was found between exposure to night-time aircraft noise and ‘heart disease and stroke’ in people who had lived in the same home for 20 years or more, and this association was robust to adjustment for exposure to NO_2_ air pollution in a subsample. An association was also found between exposure to 24 hour road traffic noise and ‘heart disease and stroke’ , but a subsample analysis suggested that this was confounded by exposure to NO_2_ air pollution.

The few studies [8-10] that have examined aircraft noise in relation to heart disease and stroke have had mixed findings, but much lower percentages of the populations in these studies experienced high (> 55 dB(A)) aircraft noise exposures than in the present study. A study of the Swiss national cohort found an effect of aircraft noise L_DN_ (weighted 24-hour average) on MI but not stroke mortality [8]. Consistent with the present analyses, the association with MI was only statistically significant in subjects who had lived for more than 15 years in the same place (hazard ratio: 1.48 (1.01, 2.18) for ≥ 60 dB(A) vs. < 45 dB(A)) [8]. A cohort study in Denmark [10] of individuals aged 50–64 years did not find an effect of aircraft noise on stroke and a cohort study in Vancouver [9] of individuals aged 45–85 years did not find an association with coronary heart disease (CHD) mortality. Other evidence relating to the association between cardiovascular disease and aircraft noise comes from a cross-sectional survey around Schiphol airport, which found an association between aircraft noise level and use of cardiovascular medication [32] and earlier studies around Schiphol, which found increased risks 7of hypertension and consumption of cardiovascular drugs and more frequent visits to doctors for cardiovascular complaints [33-35]. However these studies did not take length of residence or exposure to air pollution into account.

The significant association found in our study between aircraft noise and ‘heart disease and stroke’ in those with long residence time is more consistent with a cumulative effect of noise over time, as was found in a study of occupational noise exposure [36], than with potential habituation to noise exposure. The association between aircraft noise in the daytime and ‘heart disease and stroke’ was close to null in this study. This could be due to misclassification of exposure as participants might be away from their homes, or it may be that aircraft noise at night affects sleep and this is a potential mechanism for the observed associations. There is evidence of a link between environmental night noise and both sleep disturbance and insomnia-like symptoms [37]. Taken together with evidence from sleep laboratory experiments on the impact of arousals and lack of sleep on cardiovascular risk factors [38,39], it is plausible that lack of sleep may mediate the association between aircraft noise at night and heart disease and stroke. Aircraft noise has also been strongly related to annoyance [1] which could lead to activation of the sympathetic nervous system [22]. It has been found that exposure to road traffic noise leads to lower levels of annoyance compared to aircraft noise [40], which may partly explain the weaker association we found between road traffic noise and heart disease and stroke as compared to the association with aircraft noise at night. It is also possible that noise induces an autonomic response through the auditory pathway irrespective of any subjective reaction to noise. The field study conducted as part of the HYENA programme showed that increases in blood pressure in relation to noise events during night-time occurred even when participants reported they were asleep [20] and another HYENA study found that the association between noise and cortisol levels in women were not dependant on their degree of annoyance [21].

Data on air pollution co-exposures at a comparable spatial resolution to that for road traffic noise were available for three countries. The results from this subsample analysis suggested that associations between road traffic noise and ‘heart disease and stroke’ were confounded by air pollution, although the smaller number of cases increased the uncertainty of the estimates. However, the associations between aircraft noise and ‘heart disease and stroke’ did not appear to be affected by adjustment for air pollution. In relation to aircraft noise, these results are consistent with previous studies in that associations between aircraft noise and MI or CHD mortality have not been found to be confounded by exposure to air pollution [8,9]. The results regarding road traffic noise are consistent with a cohort study in the Netherlands which found the association between road traffic noise and cardiovascular mortality reduced after adjustment for black smoke and traffic intensity on the nearest road [15]. However, our results differ from four studies which found an independent effect of road traffic noise after adjustment for air pollution: cohort studies in Canada [9] and Denmark [10,14] and a case–control study in Sweden [13] found increased risks of CHD, MI and stroke in relation to traffic noise. Differences between studies on whether air pollution is confounding associations between road traffic noise and cardiovascular disease [18] may result from differences in the local characteristics of study areas, given that the spatial correlation between noise and air pollution is influenced by urban design features and local meteorological conditions [16,41,42].

Air pollution is a plausible confounder of associations between transport noise and cardiovascular disease given the extensive evidence of associations with long-term exposure to air pollution [17]. A statistically significant association was found in the UK and Netherlands sample between NO_2_ air pollution and ‘heart disease and stroke’. The point estimate was higher than has been found in other air pollution studies [17] but the small sample and random error must be considered in the interpretation. However, given that transport is a source of both noise and air pollutants and that noise and air pollution exposure models include the same inputs (such as traffic flows, traffic composition and traffic speed), potential for collinearity needs to be carefully considered. In this study, we used a number of statistical tests to help assess this. While collinearity was not found in this data, the two exposures come from the same source and therefore collinearity should be assessed in future studies.

This study suggests that age may be a modifier of the association between road traffic noise and ‘heart disease and stroke’ , because an association was found for those aged over 65 years. However, since the association with road traffic noise appeared to be confounded by air pollution in the subsample analysis, age as a modifier needs to be investigated in larger studies with air pollution exposures and the power to consider effect modification by age. Previous studies which have adjusted for air pollution have conflicting results on age: road traffic noise was associated with increased risk of stroke and MI in older (> 64.5 years) but not younger participants in a large Danish cohort [10,14], but age has not been found to be an effect modifier in other studies [13,15].

No sex differences were found in the association between noise and ‘heart disease and stroke’ , which may be due to lack of power given the relatively small number of cases. However previous findings on sex differences, in relation to ‘heart disease and stroke’ , have varied between studies with some reporting greater risks for men [2,8,10,12] and others not [13,15].

Strengths of this study are that it encompasses six countries from across Europe, including Italy and Greece, which have not had major studies before on this topic and that it examines not only road traffic noise but also aircraft noise, which has been little studied in relation to heart disease and stroke previously. The sampling was designed to obtain a greater proportion of participants exposed to high aircraft noise levels, which has not been possible in other studies. The study was also able to take into account multiple cardiovascular risk factors. A further strength is the inclusion of exposure to air pollution for a subsample, which suggested potential confounding of road traffic noise by air pollution. Unfortunately, data were not available to assess exposure to air pollution for all HYENA participants, which would have provided more power for the analysis.

A limitation of this study is the cross-sectional design which does not allow for causal inference. However, cases were limited to participants who had been diagnosed whilst living at their current address. Additionally, participants were only selected for inclusion in the HYENA study if they had lived for more than five years at their current address, thereby excluding people who might have moved to the exposed areas recently and already be suffering from cardiovascular disease. We did not have access to exposure data prior to 2002 and therefore some diagnoses will have been made prior to exposure. Spatial contrasts in exposure in Europe have not changed markedly over the relevant period [43] and while traffic intensities may have increased, the effect on noise levels would be modest since even a doubling of traffic volume would translate into an approximate increase of 3 dB. Given the range of noise levels in the study, any exposure misclassification is therefore unlikely to have affected the observed associations. Reliance on self-reported conditions might introduce some error because there might be over- or under-reporting [44,45]. However studies on the reliability of self-reports in comparison to medical records have found a greater concordance for well-defined conditions such as MI or stroke, which tend to have abrupt onset [46]. The analysis was conducted combining heart disease and stroke, which have some similarities but also differences in their pathogenesis. Noise has been shown to affect risk of hypertension as well as other risk factors for both heart disease and stroke [37,47]. Therefore it was thought reasonable to combine these outcomes. Moreover, when the outcomes were separated in a sensitivity analysis, the odds ratios were not materially different from the main analysis.

A possible weakness is the low response rate in most countries, which may have biased the observed results. However, for an overestimation of the associations to have occurred, it would be necessary for residents in poor health and exposed to high noise levels to have been more likely to respond than others in the area in which they lived, but no differences were found in exposure to aircraft noise between responders and non-responders [2]. Low response to a non-response survey meant that it was not possible to conduct a statistical analysis of non-response. However, it appeared that in Germany and Italy the health of the responders was slightly worse than that of the non-responders but in the Netherlands the opposite was true. Residual confounding by socio-economic status may have affected the observed findings. Individual education level was adjusted for but other indicators of socioeconomic status such as income or area-level deprivation were not collected.

## Conclusions

The findings from this cross-sectional study, together with accumulating evidence for associations between noise and hypertension [1,47] lend some support to the hypothesis that long-term exposure to aircraft noise may increase the risk of cardiovascular disease other than hypertension. However, associations between road traffic noise and cardiovascular disease may be confounded by air pollution and this should be carefully considered in future noise and health studies.

## Abbreviations

CHD: Coronary heart disease; MI: Myocardial infarction; HYENA: Hypertension and exposure to noise near airports; dB(A): A-weighted decibels; LRT: Likelihood ratio test; NO2: Nitrogen dioxide; VIF: Variance inflation factor.

## Competing interests

AH declares a potential competing interest of consultancy work in 2012 for Defra on ‘Identification of SOAEL and LOAEL (Significant/Lowest Observed Adverse Effect Level) in Support of the Noise Policy Statement for England’.

## Authors’ contributions

SF planned and carried out the analysis and drafted the manuscript. AH, CC and MB planned the analysis and contributed to the manuscript. KdH and WS carried out the mapping of noise and air pollution data to participants’ addresses. WB, DH, GP, KK, MV, FVT and EC participated in the design and coordination of the HYENA project and helped to draft the manuscript. All authors read and approved the final manuscript.

## Supplementary Material

Additional file 1 Exposure to aircraft and road traffic noise and associations with heart disease and stroke in six European countries: a cross-sectional study. Click here for file
