# Supplementary material for: Exposure to aircraft and road traffic noise and associations with heart disease and stroke in six European countries: a cross-sectional study
Source: Environ Health. 2013 Oct 16;12:89. doi: 10.1186/1476-069X-12-89 (PMC4015897; doi:10.1186/1476-069X-12-89)
Supplement: Additional file 1 — Exposure to aircraft and road traffic noise and associations with heart disease and stroke in six European countries: a cross-sectional study. [file 1476-069X-12-89-S1.doc]

**Additional file 1: Exposure to aircraft and road traffic noise and associations with heart disease and stroke in six European countries: a cross-sectional study**

Figure S1. Distributions of exposure to daytime aircraft noise LAeq,16h (cut off >= 35dB(A)), night-time aircraft noise Lnight (cut off >=30dB(A)) and 24 hour road traffic noise LAeq,24h (cut off >=45dB(A)) for HYENA participants by country


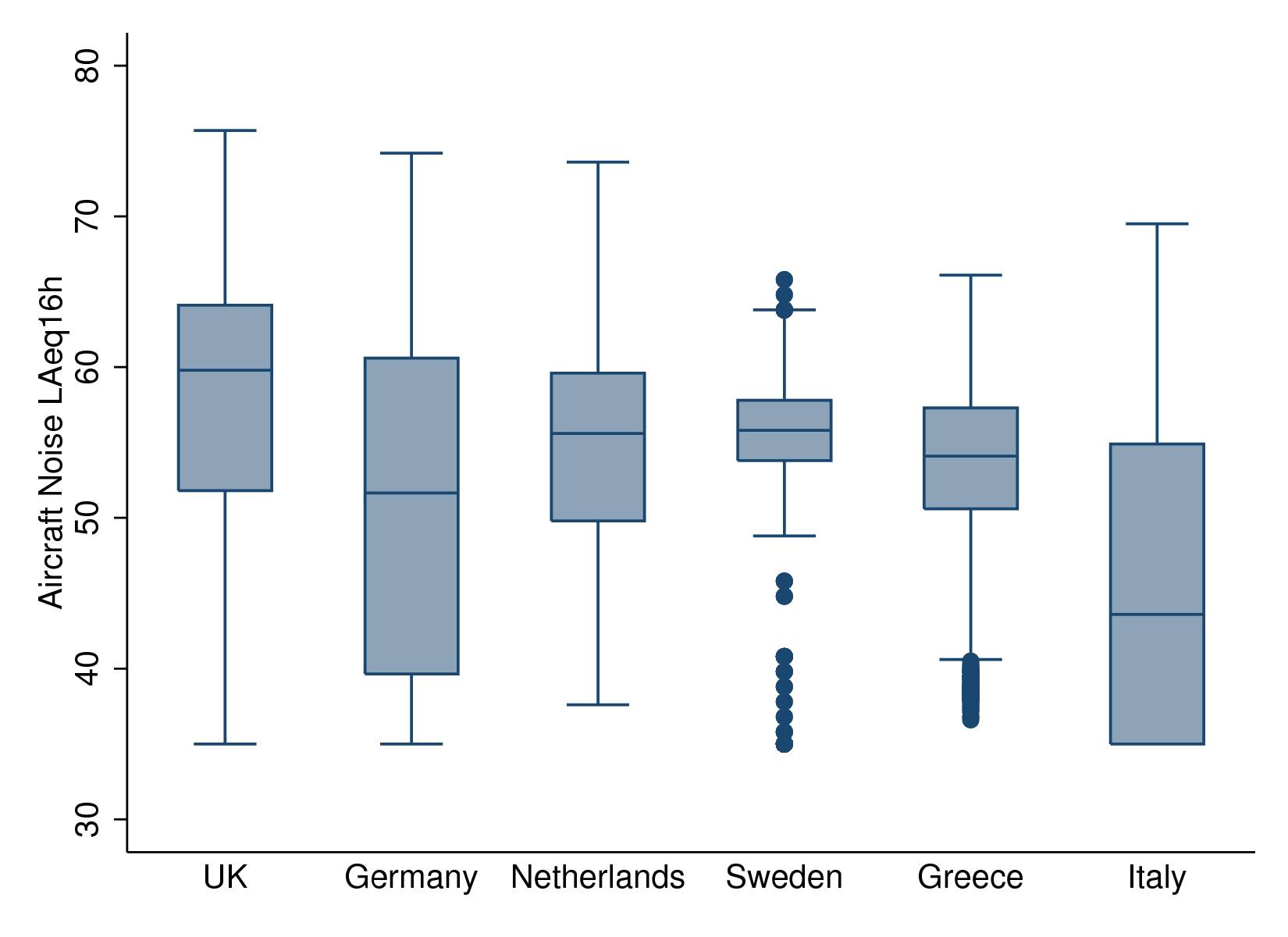

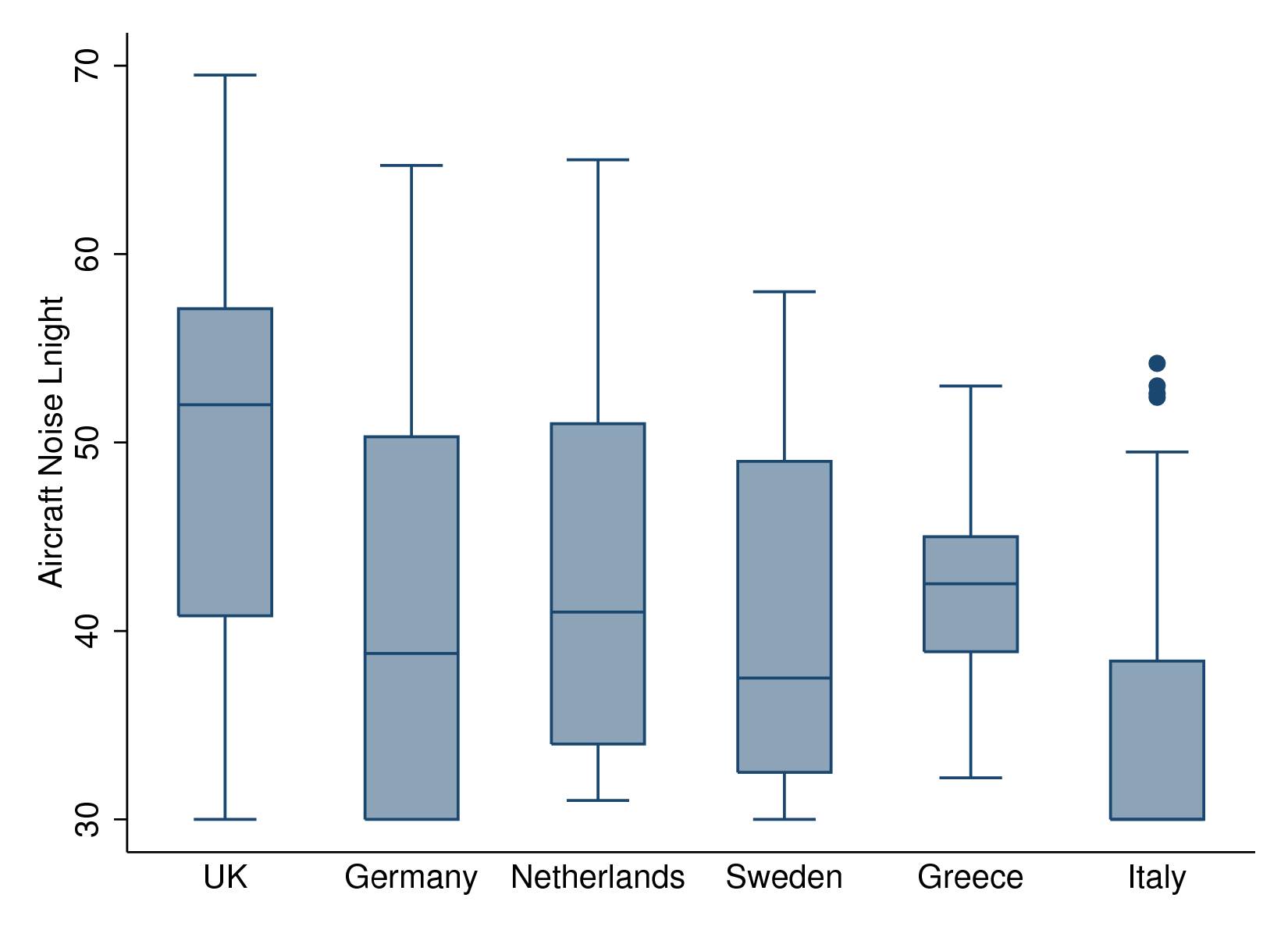

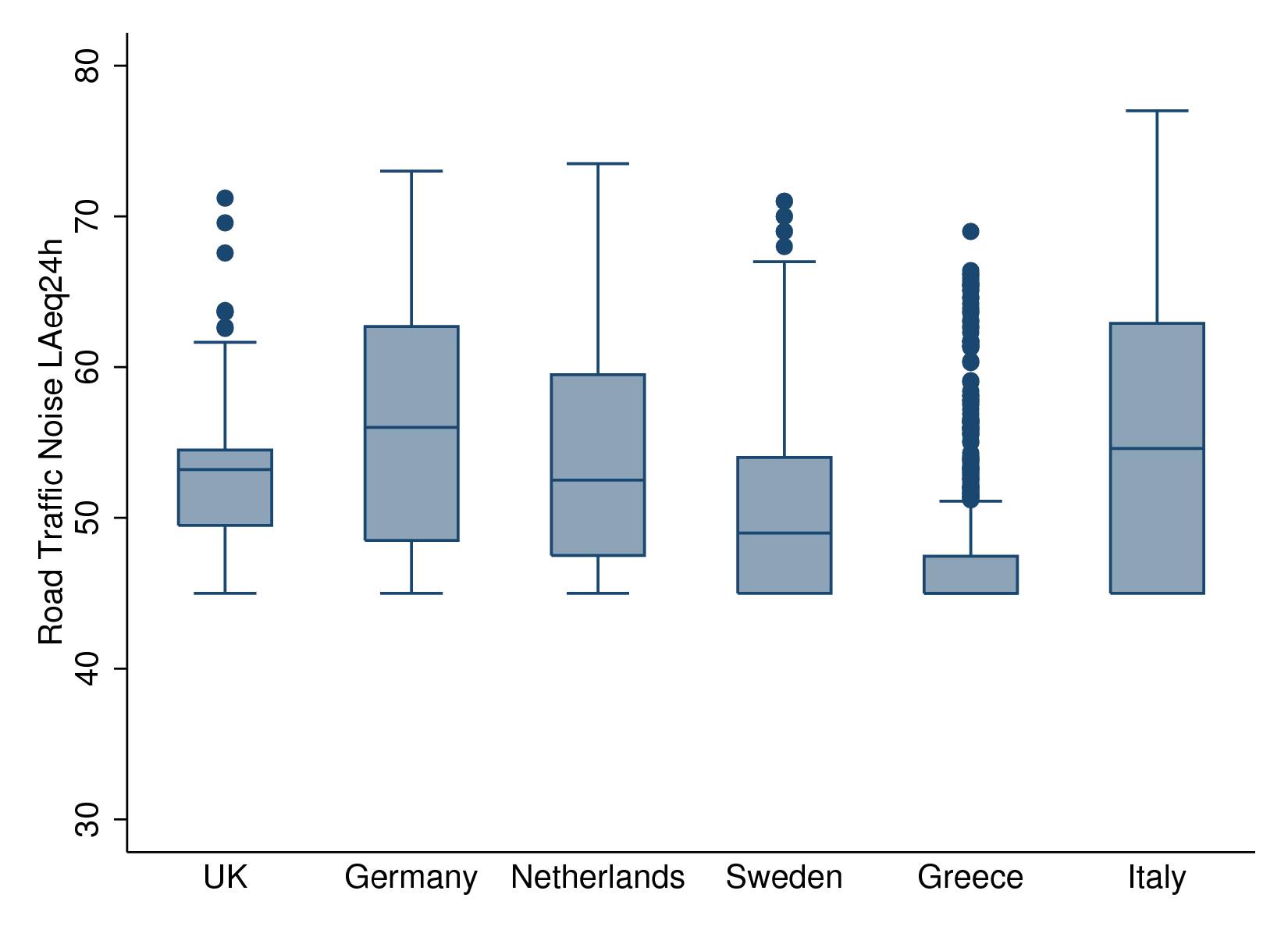


Boxplots represent the median (horizontal line), interquartile range (box), adjacent values which are the most extreme values within 1.5IQR of the nearer quartile (whiskers bounded by adjacent lines), outliers (dots)

**Exposure assessment of air pollution**

For the UK sample, concentrations of NO_2_ (μg/m^3^) for 2002 at a resolution of 20m x 20m were modelled by the Environmental Research Group (ERG) at King’s College London. These concentrations were derived using the King’s College London Emissions Toolkit and London Air Pollution Toolkit . The emissions toolkit simulates exhaust emissions from hourly road traffic flows and speeds for each of the individual roadway lengths or links in the entire London road network using a set of databases with vehicle stock and age profiles and emission factors for different types of vehicles . The King’s College London Air Pollution Toolkit was then used to model and predict the annual mean ambient concentrations of NO_2_ (μg/m^3^) using a combined modelling and measurement approach . This uses a kernel modelling technique to describe the initial dispersion. Each kernel was created using hourly meteorological data but was applied to the emissions sources as an annual mean. The model assumes three principal source types: road, rail, and gas sources which at nearby locations require detailed treatment, plus the combined emissions from more distant locations which are modelled as shallow volume sources of varying dimension. The contribution of each source is then calculated through multiple regression using annual mean concentrations at over 30 monitoring sites. The resultant modelled concentrations were linked to the HYENA participants’ home addresses using GIS methods.

For the Netherlands sample, NO_2_ concentrations were provided for 2005 at a spatial resolution of 25m x 25m. The EMPARA Luvotool model used to calculate air pollution for the Netherlands had three parts: urban roads, non-urban roads and background (e.g. large scale contributions) . Emission factors were applied for 5 speed classes: motorway, non-urban road, urban traffic (less congestion), normal urban traffic, stagnating urban traffic. For the urban roads, Luvotool used a dispersion model calibrated to measurements. The model included five road types and took into account land use information to determine the rate of dilution. Wind speed and presence of trees were also included. For the non-urban roads, Luvotool used a Gaussian plume dispersion model (VLW) which was suitable for areas without significant obstacles such as buildings and tall trees. This model took account of both wind speed and wind direction. For the background concentrations, the OPS dispersion model was used (with a resolution of 1km x 1km) which used data on emissions from the Netherlands and abroad.

The air pollution modelling for the Swedish participants was carried out by SLB-analys, using the emission databases and dispersion models of Stockholm and Uppsala Air Quality Management Association . They provided annual mean concentrations of NO_2_ for 2002 for each HYENA participant’s address. This included emissions from road traffic and from the airport. The airport emissions were provided as annual mean values of NO_x_ by the Swedish Airports and Air Navigation Services. Annual mean total concentrations were calculated using two levels of geographic resolution, combining large scale calculations with coarse resolution with high resolution calculations (20m x 20m). Meteorological data for 2002 was used. Non-local sources were also considered by adding rural background concentrations from monitoring data. Dispersion of the pollutants from the sources was estimated with a dilution model based on the average annual distribution of wind speed, direction and precipitation, using the Airviro dispersion model. Other inputs into the model included ground contours, ground surface type, building density and building height.

These air pollution models have been validated against measurements

References:

Table S1. Spearman’s ρ correlations of noise exposures for HYENA participants

|  | Daytime aircraft noise | Night-time aircraft noise | 24 hour road traffic noise |
| --- | --- | --- | --- |
|  |  |  |  |
| Daytime aircraft noise | 1 | 0.82 | 0.01 |
| Night-time aircraft noise | - | 1 | 0.02 |
| 24 hour road traffic noise | - | - | 1 |

Figure S2. Distributions of Exposure to Road Traffic Noise L_aeq,24h_ (cut off >=45dB(A)) and Nitrogen Dioxide (µg/m^3^) in HYENA project, UK, Netherlands and Sweden, 2004-2006


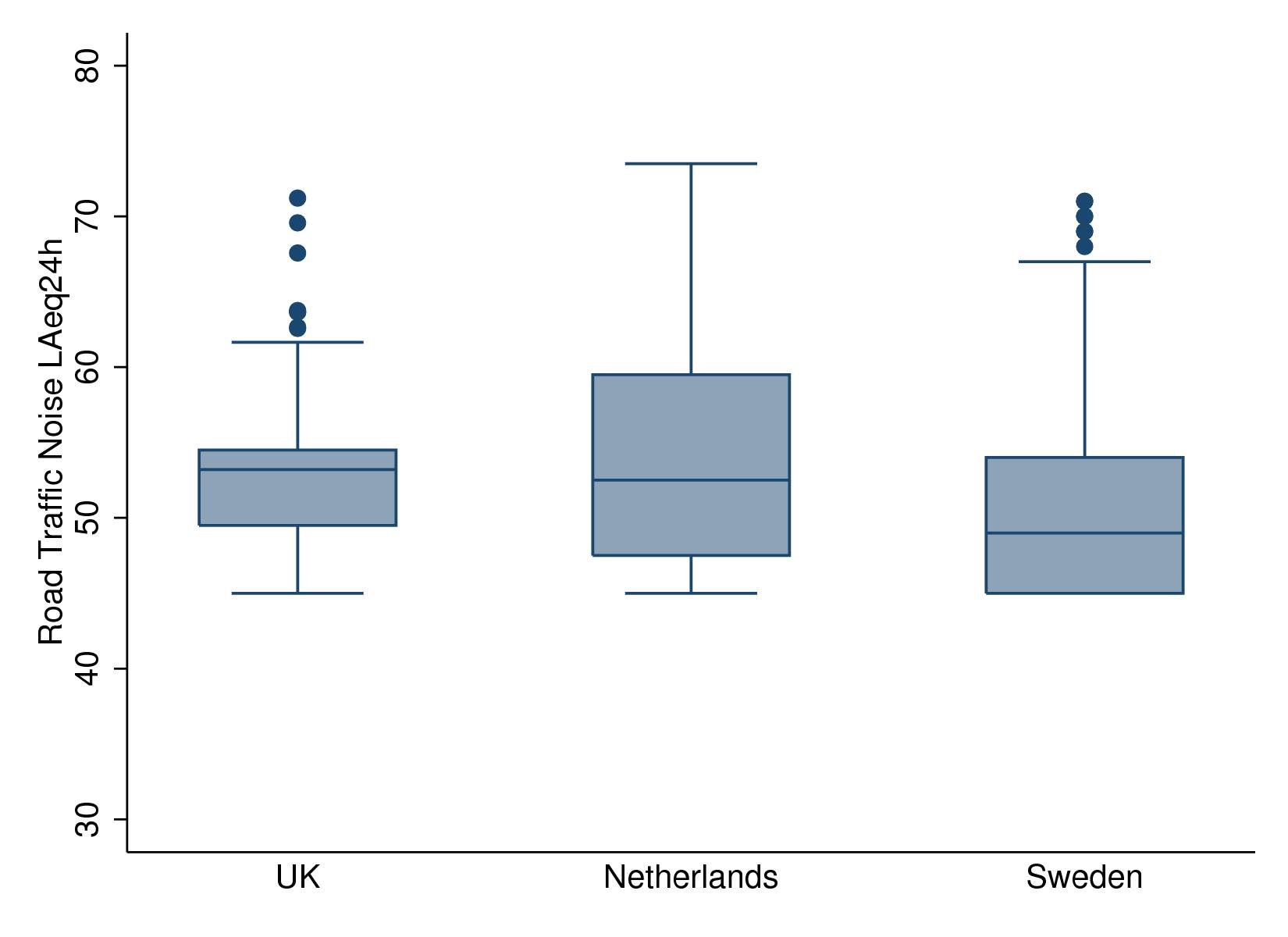

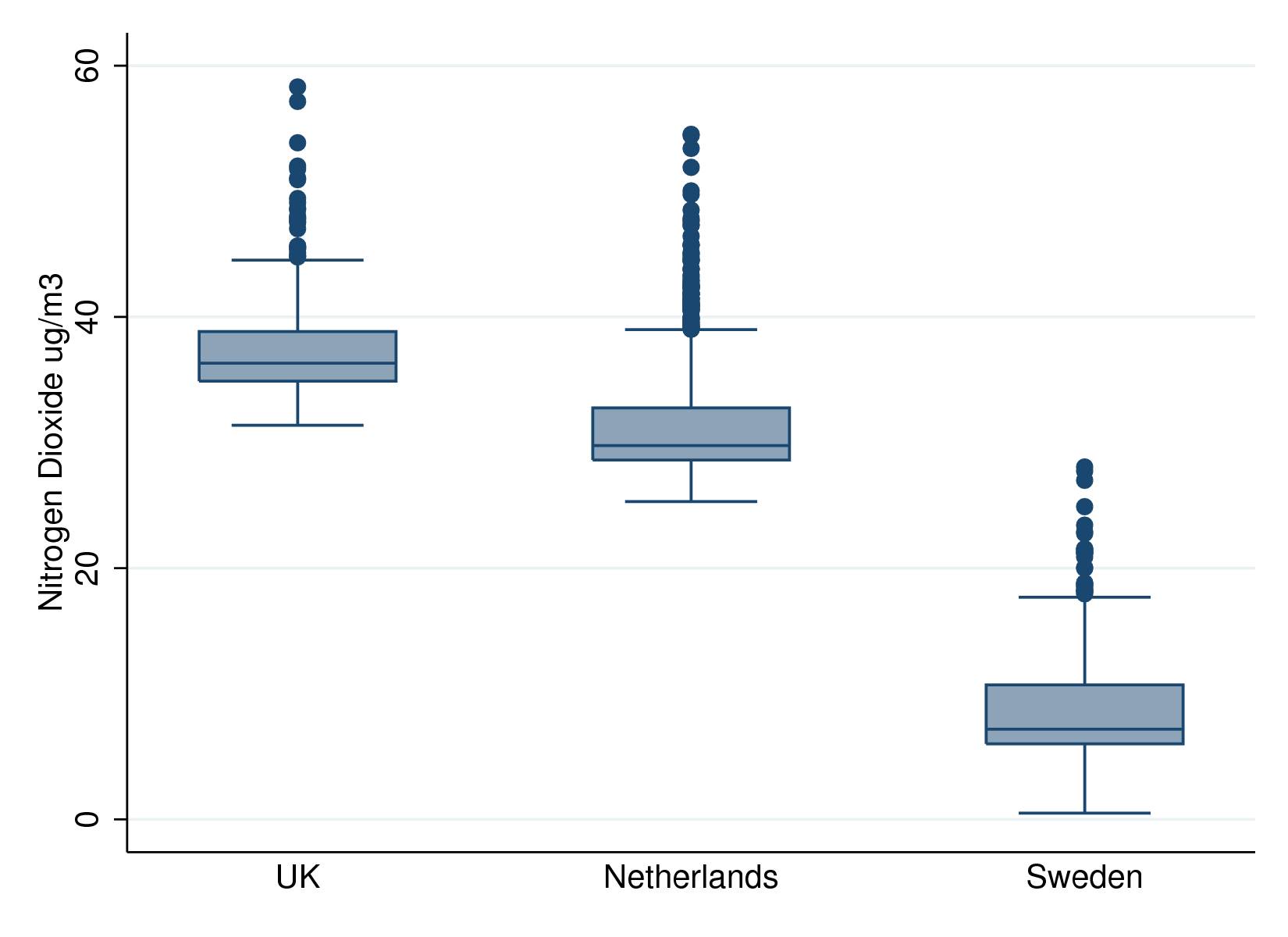


Boxplots represent the median (horizontal line), interquartile range (box), adjacent values which are the most extreme values within 1.5IQR of the nearer quartile (whiskers bounded by adjacent lines), outliers (dots)

**Figure S3. HYENA sample sizes**

**
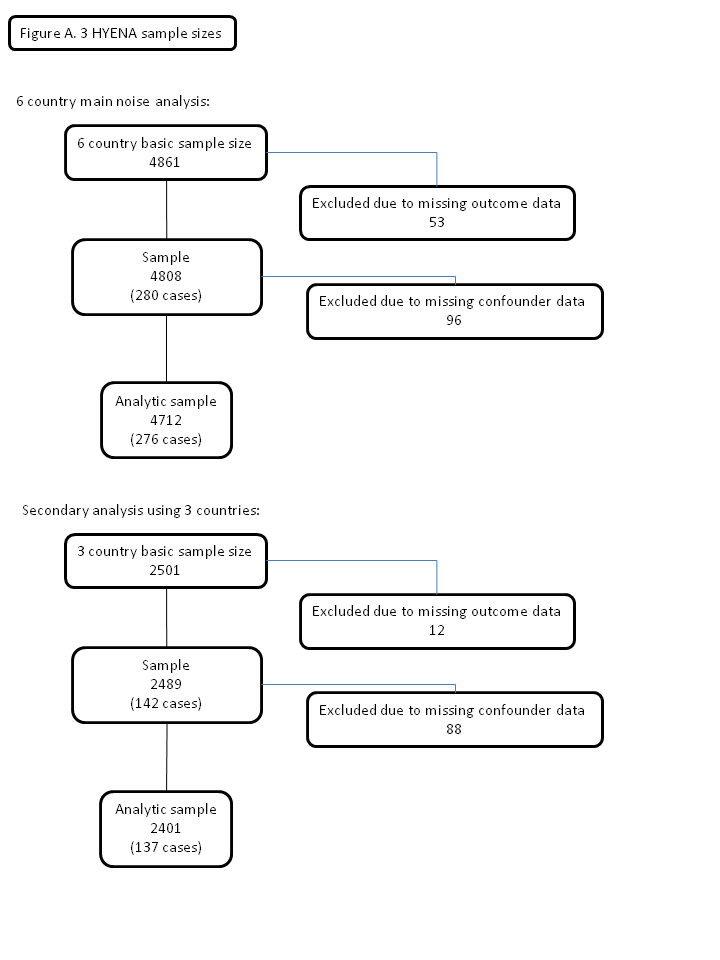
**

Table S2. Frequency distribution of exposure to noise of study population (n = 4712)

| **Exposure** | **N** | **%** |
| --- | --- | --- |
| Daytime aircraft noise (dB(A)) |  |  |
| <45 | 1182 | 25.1 |
| 45 – 49.9 | 384 | 8.2 |
| 50 – 54.9 | 908 | 19.3 |
| 55 – 59.9 | 1283 | 27.2 |
| 60 – 64.9 | 735 | 15.6 |
| >=65 | 220 | 4.7 |
| Night-time aircraft noise (dB(A)) |  |  |
| <35 | 1709 | 36.3 |
| 35 – 39.9 | 659 | 14.0 |
| 40 – 44.9 | 603 | 12.8 |
| 45 – 49.9 | 797 | 16.9 |
| 50 – 54.9 | 588 | 12.5 |
| >= 55 | 356 | 7.6 |
| 24 hour road traffic noise (dB(A)) |  |  |
| <45 | 1117 | 23.7 |
| 45 – 49.9 | 1050 | 22.3 |
| 50 – 54.9 | 922 | 19.6 |
| 55 – 59.9 | 730 | 15.5 |
| 60 – 64.9 | 473 | 10.0 |
| >=65 | 420 | 8.9 |

**Figure S4. Associations (ORs and 95% CIs) of ‘heart disease and stroke’ in relation to night-time aircraft noise in 5dB(A) categories (reference category: <=34.9dB(A)). Model included random intercept for country and adjusted for ethnicity, age, education, sex, BMI and 24 hour road traffic noise**


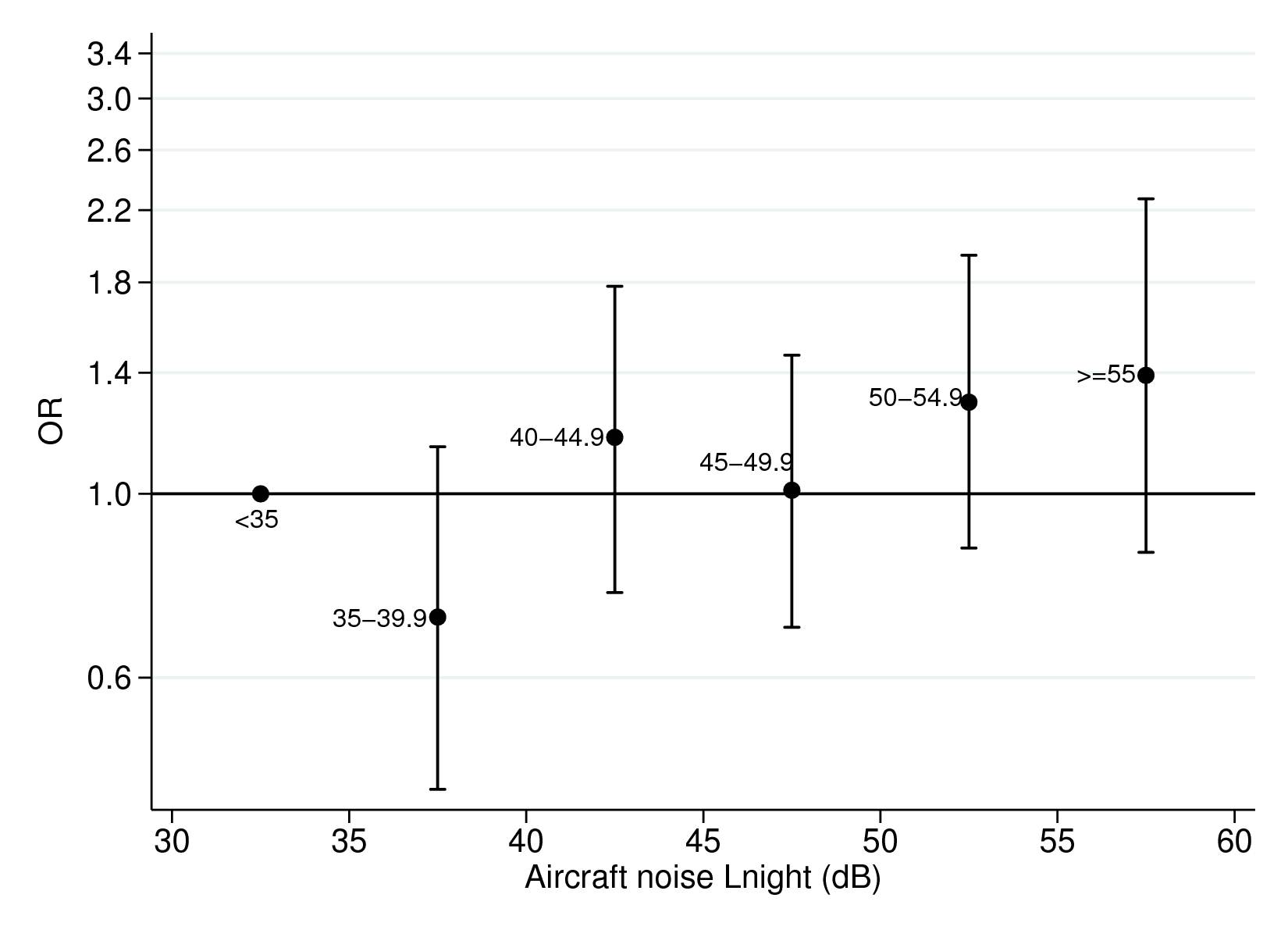


Number of cases in each category:

<35dB: 91; 35-39.9dB: 25; 40-44.9dB: 38; 45-49.9dB: 46; 50-54.9dB: 44; >=55dB: 32

Figure S5. Associations (ORs and 95% CIs) of ‘heart disease and stroke’ in relation to 24 hour road traffic noise in 5dB(A) categories (reference category: <=47.4dB(A)). Model included random intercept for country and adjusted for ethnicity, age, education, sex, BMI and night-time aircraft noise


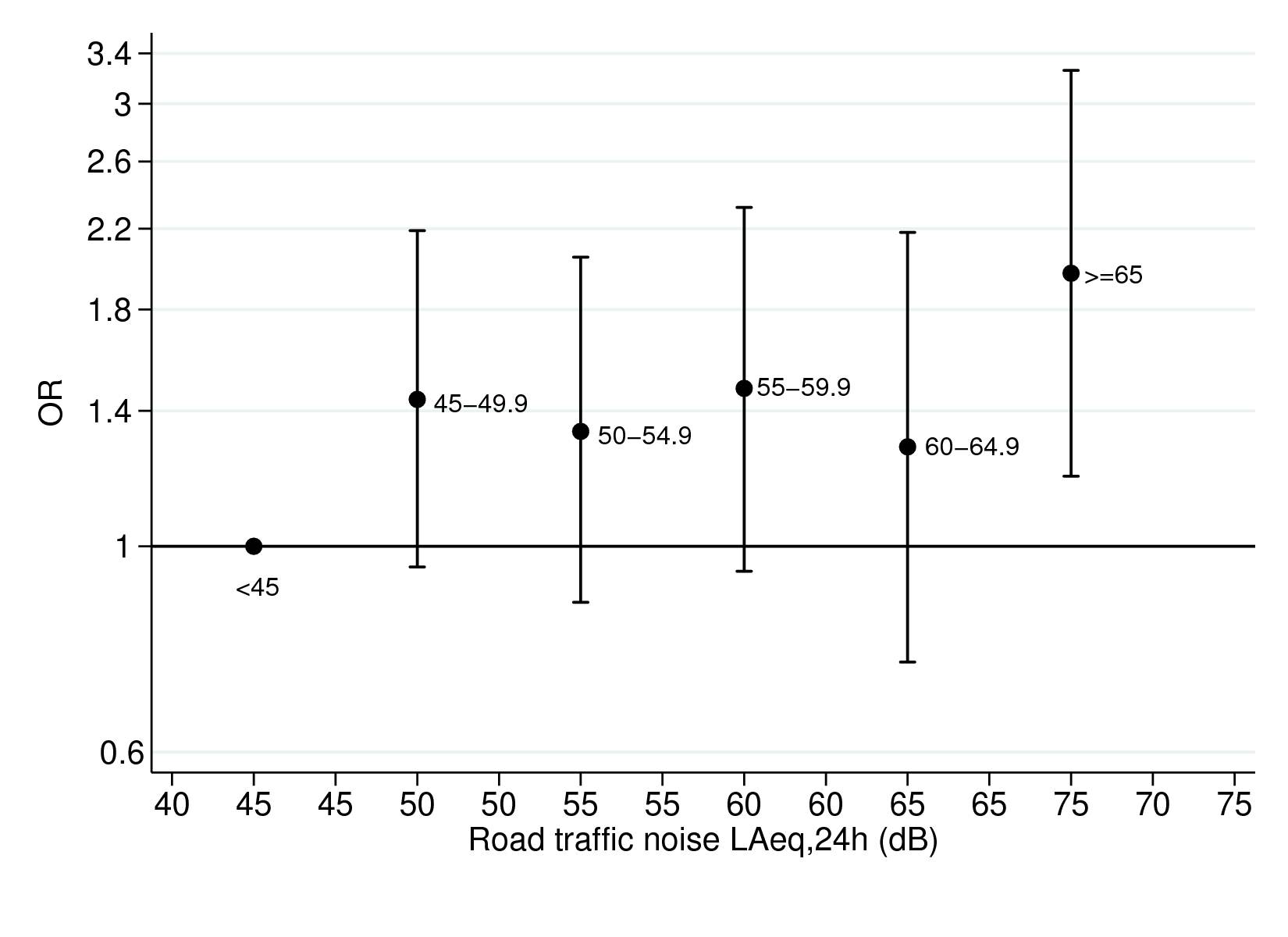


Number of cases in each category:

<45dB: 47; 45-49.9dB: 66; 50-54.9dB: 58; 55-59.9dB: 48; 60-64.9dB: 25; >=65dB: 32

TableS3. Associations between heart disease and stroke, as separate outcomes, and daytime aircraft noise, night-time aircraft noise and 24-hour road traffic noise

Odds ratios and 95% Confidence Intervals

| **Heart disease (myocardial infarction or angina pectoris)**  **Participants = 4712; Cases = 229** | **Daytime aircraft noise per 10dB(A)** | **Night-time aircraft noise per 10dB(A)** | **24 hr road traffic noise per 10dB(A)** |
| --- | --- | --- | --- |
| Crude (exposure and random intercept^a^) | 1.12 (0.96, 1.30) | 1.22 (1.05, 1.42) | 1.26 (1.05, 1.52) |
| Adjusted for age, sex, BMI, education, ethnicity^b^ | 1.06 (0.91, 1.22) | 1.11 (0.96, 1.29) | 1.23 (1.03, 1.46) |
| Adjusted for age, sex, BMI, education, ethnicity and other noise exposures^c^ | 1.06 (0.92, 1.23) | 1.12 (0.97, 1.30) | 1.24 (1.03, 1.48) |
| **Stroke**  **Participants = 4712; Cases = 62** |  |  |  |
| Crude (exposure and random intercept^a^) | 1.07 (0.82, 1.39) | 1.17 (0.89, 1.55) | 1.08 (0.76, 1.53) |
| Adjusted for age, sex, BMI, education, ethnicity^b^ | 1.08 (0.82, 1.41) | 1.18 (0.89, 1.56) | 1.06 (0.75, 1.51) |
| Adjusted for age, sex, BMI, education, ethnicity and other noise exposures^c^ | 1.08 (0.82, 1.41) | 1.18 (0.89, 1.57) | 1.07 (0.75, 1.52) |

Table S4. Subsample analysis: Indicators of collinearity between noise and air pollution in UK and Netherlands separately

Correlations of Exposure Variables (Spearman’s ρ), Correlation of Estimated Coefficients of Exposure Variables, Variance Inflation Factor

| **Collinearity indicators^a^** | **Daytime aircraft noise per 10dB(A)** | **Night-time aircraft noise per 10dB(A)** | **24 hr road traffic noise per 10dB(A)** |
| --- | --- | --- | --- |
| **UK** |  |  |  |
| **Participants = 548** |  |  |  |
| Spearman’s ρ | -0.06 | -0.07 | 0.53 |
| Correlation of estimated coefficients | 0.18 | 0.21 | -0.47 |
| Variance Inflation Factor | 1.00 | 1.00 | 1.30 |
| **Netherlands** |  |  |  |
| **Participants = 864** |  |  |  |
| Spearman’s ρ | -0.04 | -0.09 | 0.74 |
| Correlation of estimated coefficients | 0.31 | 0.28 | -0.67 |
| Variance Inflation Factor | 1.02 | 1.03 | 1.87 |

** P* < 0.05

***P* < 0.0001

^a^ Only the two exposures were considered, adjustment was not made for other covariates

Table S5. Subsample analysis: associations between ‘heart disease and stroke’ and noise adjusted for exposure to nitrogen dioxide, in UK and Netherlands separately.

Associations expressed in odds ratios and 95% confidence intervals

| **Heart disease and stroke** | **Daytime aircraft noise per 10dB(A)** | **Night-time aircraft noise per 10dB(A)** | **24 hr road traffic noise per 10dB(A)** |
| --- | --- | --- | --- |
| **UK** |  |  |  |
| **Participants = 547; Cases = 48** |  |  |  |
| Crude (adjusted for country) | 1.23 (0.88, 1.72) | 1.17 (0.87, 1.56) | 1.10 (0.63, 1.94) |
| Adjusted ^a^ | 1.02 (0.71, 1.45) | 1.00 (0.73, 1.38) | 1.03 (0.56, 1.92) |
| Adjusted ^a^ plus nitrogen dioxide exposure | 1.08 (0.75, 1.57) | 1.08 (0.77, 1.50) | 0.60 (0.28, 1.31) |
| **Netherlands** ^b^ |  |  |  |
| **Participants = 864; Cases = 36** |  |  |  |
| Crude | 1.51 (0.86, 2.63) | 1.30 (0.90, 1.87) | 1.48 (0.94, 2.34) |
| Adjusted ^a^ | 1.74 (0.95, 3.19) | 1.36 (0.92, 2.02) | 1.54 (0.93, 2.54) |
| Adjusted ^a^ plus nitrogen dioxide exposure | 1.83 (0.98, 3.41) | 1.45 (0.96, 2.20) | 1.25 (0.63, 2.50) |

^a^Adjusted for age, sex, education, ethnicity, BMI, physical activity (<once/week, 1-3 times/week, >3 times/week), smoking (never, past, current), alcohol intake (teetotal, 1-7 units/week, 8-14 units/week, >14 units/week; 1 unit = 10 ml pure ethanol). In addition, the aircraft noise models were adjusted for 24 hour road traffic noise and the road traffic noise model was adjusted for night-time aircraft noise

^b^Ethnicity was not included in the models when the analysis was concentrated only on the Netherlands sample because there were no cases of CVD in the non-white group.

Table S6. Subgroup analysis: associations (odds ratios and 95% confidence intervals) between ‘heart disease and stroke’ and nitrogen dioxide in HYENA project, UK and Netherlands combined and Sweden separately, 2004-2006

| Heart disease and stroke | Nitrogen Dioxide  per 10µg/m^3^ |
| --- | --- |
| UK and Netherlands combined |  |
| Participants =1411; Cases = 84 |  |
| Crude (adjusted for country) | 1.75 (1.11, 2.74) |
| Adjusted ^a^ | 1.85 (1.13, 3.02) |
| Adjusted ^a^ plus road traffic noise exposure | 1.95 (1.03, 3.70) |
| % change in coefficient (absolute value) | 9% |
| Sweden |  |
| Participants=990; Cases = 53 |  |
| Crude | 1.07 (0.53, 2.18) |
| Adjusted ^a^ | 1.39 (0.62, 3.10) |
| Adjusted ^a^ plus road traffic noise exposure | 1.40 (0.58, 3.38) |
| % change in coefficient (absolute value) | 3% |

^a^ Adjusted for age, sex, education, ethnicity, BMI, physical activity, smoking, alcohol intake, night-time aircraft noise
